# Moderate Vaccine Effectiveness against Severe Acute Respiratory Infection Caused by A(H1N1)pdm09 Influenza Virus and No Effectiveness against A(H3N2) Influenza Virus in the 2018/2019 Season in Italy

**DOI:** 10.3390/vaccines8030427

**Published:** 2020-07-30

**Authors:** Caterina Rizzo, Francesco Gesualdo, Daniela Loconsole, Elisabetta Pandolfi, Antonino Bella, Andrea Orsi, Giulia Guarona, Donatella Panatto, Giancarlo Icardi, Christian Napoli, Giovanni Battista Orsi, Ilaria Manini, Emanuele Montomoli, Ilaria Campagna, Luisa Russo, Valeria Alfonsi, Simona Puzelli, Antonino Reale, Umberto Raucci, Livia Piccioni, Carlo Concato, Marta Luisa Ciofi Degli Atti, Alberto Villani, Maria Chironna, Alberto Eugenio Tozzi

**Affiliations:** 1 IRCCS, Bambino Gesù Children’s Hospital, 00165 Rome, Italy; francesco.gesualdo@opbg.net (F.G.); elisabetta.pandolfi@opbg.net (E.P.); ilaria.campagna@opbg.net (I.C.); luisa.russo@opbg.net (L.R.); antonino.reale@opbg.net (A.R.); umberto.raucci@opbg.net (U.R.); livia.piccioni@opbg.net (L.P.); carlo.concato@opbg.net (C.C.); marta.ciofidegliatti@opbg.net (M.L.C.D.A.); alberto.villani@opbg.net (A.V.); albertoeugenio.tozzi@opbg.net (A.E.T.); 2 Department of Biomedical Science and Medical, Oncology of the University of Bari, 70120 Bari, Italy; daniela.loconsole@uniba.it (D.L.); maria.chironna@uniba.it (M.C.); 3 Department of Infectious Diseases, National Institute of Health, 00161 Rome, Italy; antonino.bella@iss.it (A.B.); simona.puzelli@iss.it (S.P.); 4 IRCCS University Hospital San Martino, 16100 Genoa, Italy; andrea.orsi@unige.it (A.O.); 3479731@studenti.unige.it (G.G.); donatella.panatto@unige.it (D.P.); icardi@unige.it (G.I.); 5 Department of Medical-Surgical Sciences and Translational Medicine, University of Rome “Sapienza”, 00185 Rome, Italy; christian.napoli@uniroma1.it; 6 Department of Public Health and Infectious Diseases, Sapienza University of Rome, 00185 Rome, Italy; giovanni.orsi@uniroma1.it (G.B.O.); ilaria.manini@unisi.it (I.M.); 7 Department of Molecular and Developmental Medicine, University of Siena, 53100 Siena, Italy; emanuele.montomoli@unisi.it; 8 Vaccine Assessment VisMederi Srl, 53100 Siena, Italy; 9 Medical Direction, University Hospital Sant’Andrea, 00189 Rome, Italy; valfonsi@ospedalesantandrea.it

**Keywords:** influenza vaccine effectiveness, test-negative case-control study, SARI, Italy, IT-BIVE-HOSP network

## Abstract

Every season, circulating influenza viruses change; therefore, vaccines must be reformulated each year. We aimed to estimate vaccine effectiveness (VE) against severe influenza infection for the 2018/19 season in Italy. We conducted a test-negative design case-control study at five Italian hospitals. We estimated influenza VE against severe acute respiratory infection (SARI) requiring hospitalisation overall, and by virus subtype, vaccine brand, and age. The 2018/19 season was characterised by A(H1N1)pmd09 and A(H3N2) influenza viruses. Vaccine coverage among <18 years recruited SARI cases was very low (3.2%). Seasonal vaccines were moderately effective against type A influenza overall (adjusted VE = 40.5%; 95% confidence interval (CI) = 18.7–56.4%) and subtype A(H1N1)pmd09 viruses (adjusted VE = 55%; 95% CI = 34.5–69.1%), but ineffective against subtype A(H3N2) viruses (adjusted VE = 2.5%; 95% CI = −50.0–36.7%). Both Fluad and Fluarix Tetra vaccines were effective against type A influenza overall and subtype A(H1N1)pdm09 viruses. VE appeared to be similar across age groups (0–64 years, ≥65 years). Seasonal influenza vaccines in the 2018/19 season were moderately effective in preventing SARI caused by A(H1N1)pdm09 influenza but ineffective against A(H3N2).

## 1. Introduction

Influenza is a major public health burden, accounting for up to 50 million disease episodes [1] and 72,000 deaths in Europe each year [2]. In Italy alone, there were 8.7 million symptomatic cases of influenza in the 2017/18 influenza season [3].

The consequences of the influenza infection can be severe, both for the individual and for the health care system. The severity of the infection depends on the virus type/subtype, on host characteristics (e.g., age), and on other factors, such as access to care. Complications of influenza, such as pneumonia, are more common among specific risk groups, including elderly individuals, children under one year of age and people affected with immune deficiencies. Severe acute respiratory infection (SARI) caused by the influenza virus can result in hospitalisation [4].

Seasonal vaccination is considered as the most effective way to prevent influenza and its complications, with a recommended target of a 75% coverage among groups at high risk of infection [5]. In Italy, annual vaccination is provided free of charge for individuals who are high risk, including those aged ≥65 years, health care workers, people affected by chronic diseases and immune deficiencies, and pregnant women [6]. In 2018/19, around 15.8% of the Italian population overall and 53.1% of people ≥65 years received a seasonal influenza vaccine [7].

Circulating influenza viruses vary from season to season and influenza vaccines are reformulated regularly. This means that vaccine effectiveness (VE) studies must be conducted on an annual basis. In the 2017/18 influenza season, VE was estimated at 38% across nine European countries, including Italy. VE varied by age group, with the highest effectiveness among people aged 0–17 years (59%). For people aged 18–64 years and ≥65 years, VE was estimated at 34 and 44%, respectively. During the same season, over two-thirds of influenza cases were caused by type B influenza. Of the type A circulating influenza viruses, the majority were A(H1N1)pmd09 [8].

During the 2017/18 season, a study was conducted in Italy to estimate VE of seasonal influenza vaccination, focusing specifically on SARI caused by the influenza virus, but the analysis was limited to people ≥65 years receiving a trivalent vaccine [9].

With the present study, we aim to estimate the seasonal influenza VE in Italy in the 2018/2019 season, focusing on SARI caused by the influenza virus. Expanding the focus of the 2017/2018 season study, we present estimates of VE against laboratory-confirmed influenza infection requiring hospitalisation for people of all ages, overall, and stratified by vaccine brand, virus subtype, and age group.

## 2. Methods

### 2.1. Study Design

This was a multicentre, hospital-based, test-negative design case-control study. The primary objective was to measure seasonal influenza VE against laboratory-confirmed SARI requiring hospitalisation, overall, and by vaccine brand. Secondary objectives included estimating VE by age group, influenza virus types (A/B), and subtypes (A(H1N1)pdm09/A(H3N2)).

### 2.2. Study Setting and Population

Data came from five Italian hospitals participating in the IT-BIVE-HOSP network, which aims to estimate brand-specific influenza VE among hospitalised patients in Italy. The hospitals were located throughout Italy: two hospitals were in Rome and the other three were in Genoa, Siena, and Bari. The study population consisted entirely of community-dwelling individuals admitted to one of these five hospitals with a diagnosis of SARI during the 2018/19 flu season (defined as the period between the first influenza virus being detected and the point when no viruses had been detected for 2 weeks).

### 2.3. Case Finding

We used the following clinical case definition for SARI: a person presenting at the emergency department with at least one systemic symptom (fever or feverishness, malaise, headache, or myalgia), or deterioration of general condition, and at least one respiratory symptom (cough, sore throat, or shortness of breath) at admission or within 48 h after admission.

Cases and controls were identified using International Classification of Diseases (ICD-9 and ICD-10 diagnostic codes) in four of the five study hospitals. In one hospital where codes were not systematically recorded (Siena), patients were screened on admission and people meeting the clinical case definition for SARI who did not meet any exclusion criteria were invited to participate in the study. A “case” was an individual presenting with SARI, whose respiratory sample tested positive for influenza. A “control” was an individual presenting with SARI, whose respiratory sample tested negative for influenza.

Patients were excluded from the study if they met one or more of the following criteria:

- Age < 6 months at time of recruitment;
- Presence of contraindication for the influenza vaccine;
- Previously hospitalised < 48 h prior to SARI onset;
- SARI onset ≥ 48 h after hospital admission;
- Respiratory specimen taken >7 days after SARI onset;
- Tested positive for any influenza virus in the 2018/19 season before the onset of symptoms leading to the current hospitalisation;
- Vaccination ≤ 14 days before SARI symptom onset, no vaccine record for 2018/19 season, or ambiguous vaccination status;
- Unwilling to participate or unable to communicate and give consent;
- Institutionalised at the time of symptom onset (living in a residence for people who require continual nursing care and have difficulty with activities of daily living).

One patient who tested positive for type B influenza was also excluded from the analysis, as no meaningful analysis of VE against type B influenza could be conducted with data from a single subject.

### 2.4. Data Collection

Study investigators collected demographic, epidemiological, and clinical data from hospital records. Additional information (e.g., number of hospitalisations and general practitioner (GP) visits in the past 12 months) was collected using a standardised questionnaire administered during interviews with patients (and/or their relatives) before swabbing to test for the influenza virus.

Patients were asked about their vaccination status during interviews. For those responding that they were vaccinated or who did not know their status, the patient’s GP was contacted to confirm vaccination status, date of vaccination, and vaccine brand. Patients were considered vaccinated if records showed receipt of at least one dose of injectable influenza vaccine (or at least two doses for children <9 years) more than 14 days before the onset of SARI symptoms. In line with the exclusion criteria, patients were excluded from the analysis if they were partially vaccinated (vaccine administered ≤14 days before SARI symptom onset), had no record of vaccination for the current season, or if positive vaccination status could not be confirmed in GP records or was otherwise ambiguous.

### 2.5. Laboratory Analysis

Respiratory specimens were collected from all eligible SARI patients and analysed using RT-PCR and multiplex RT-PCR. The laboratories were located inside each of the participating hospitals. Isolates underwent a molecular analysis for currently circulating influenza viruses.

### 2.6. Statistical Analysis

Variables were described as mean and SD or proportions, as appropriate. Age was classified in three groups for the descriptive analysis (6 months–17 years, 18–64 years, and ≥65 years).

Baseline characteristics of cases and controls were compared using chi-squared or Fisher’s exact tests (for categorical variables), Student’s *t*-tests (for normally distributed continuous variables) or Mann–Whitney tests (for ordinal or non-normally distributed continuous variables). Missing data relating to lag time (time between onset of SARI symptoms and swab being taken) and numbers of GP visits and hospitalisations in the last year were imputed through multiple imputation.

Estimates of VE were calculated for all influenza viruses and all seasonal vaccines, then stratified by virus subtype, vaccine brand, and patient age group. For the VE estimation, only two age groups were considered (6 months–64 years and ≥65 years), given the low vaccine coverage in the 6 months–17 years age group. Crude point estimates of VE were calculated using the odds ratio (OR) of vaccination for influenza-positive SARI patients vs. influenza-negative SARI patients, with the formula *VE* = 1 − *OR*. Ninety-five percent confidence interval (95% CI) was computed around point estimates.

Multivariable logistic regression models were used to calculate confounder-adjusted VE estimates. Potential confounders were tested in the univariate analysis and those that gave *p*-values < 0.02 were included in the regression model. Potential confounders and effect modifiers of interest included sex, age group, presence of a chronic disease, lag time, month of SARI onset, and number of GP consultations and hospitalisations in the last 12 months. All statistical analyses were carried out using Stata version 13.1 (StataCorp, College Station, TX, USA).

### 2.7. Ethical Approval and Consent

Ethical approval was granted by the Ethical Committee of the Bambino Gesù Children’s Hospital (prot. N.1633_OPBG_2018). Informed written consent was obtained from all patients included in the study or, where necessary, their legal representatives.

## 3. Results

### 3.1. Patient Population

Initially, 1693 patients with SARI were identified in the five participating centres. Seventeen patients were excluded because their vaccination status was unknown, six because their laboratory sample was taken >7 days after SARI onset, and one because they were vaccinated ≤14 days before presenting with SARI. This left a cohort of 1667 patients, consisting of 1167 controls and 500 cases.

The majority of patients (1004; 60.2%) came from the hospital in Bari, while 233 (14.0%) were from the hospital in Genova, 254 (15.2%) from the Bambino Gesù Children’s Hospital in Rome, 142 (8.5%) from the Sant’Andrea Hospital in Rome, and 34 (2.0%) from the hospital in Siena.

Most patients (832, 49.9%) were younger than 18 years of age, 290 (17.4%) belonged to the 18–64 age group, and 545 (32.7%) were over 65 years of age.

Cases and controls were similar in terms of demographic characteristics (Table 1).

Statistical analysis suggested a significant difference between cases and controls in terms of smoking status (*p* = 0.000) and of presence of chronic conditions (*p* = 0.015). In addition, the mean number of visits to a GP in the past year was higher for controls (*p* = 0.015).

### 3.2. Surveillance Data

**Figure 1:**
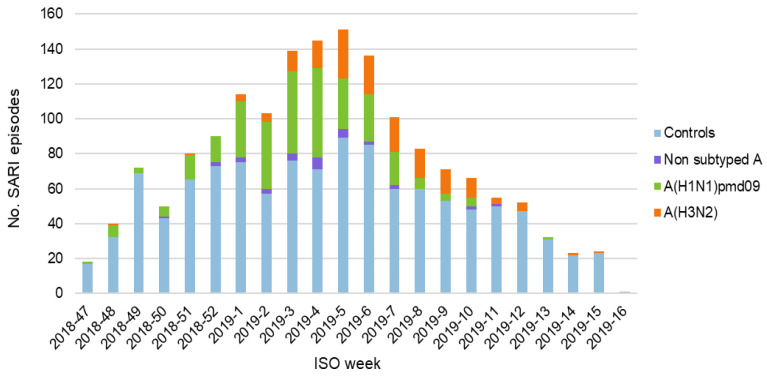
Episodes of severe acute respiratory infection (SARI) per week, stratified by cases/controls and influenza subtype.

Episodes of SARI were recorded between the 20th of November (week 47) 2018 and the 23rd of April (week 16) 2019. Both the total number of recorded episodes and the number of patients with laboratory-confirmed influenza peaked in weeks 3–6, 2019 (Figure 1).

Of the 500 cases with laboratory-confirmed influenza, 305 (61.0%) tested positive for A (H1N1) pdm09 and 162 (32.4%) for A (H3N2). Non-subtyped type A influenza accounted for 33 cases (6.6%). Only one case of type B influenza was recorded. This case was subsequently excluded because no meaningful analysis of VE against type B influenza could be conducted with data from a single subject.

### 3.3. Vaccine Coverage

Seasonal vaccine coverage was 20.7% overall (Table 2).

As expected, controls were more likely to have received a seasonal vaccine (22.3% vs. 17.0%; *p* = 0.015). Vaccine coverage among children was very low (3.2%). People aged 0–64 years were less likely to have received a vaccine than those in the older age group (6.8% vs. 49.4%), in line with Italy’s policy of free seasonal vaccination for people ≥65 years.

When coverage was stratified by vaccine brand, we found that 59.4% of vaccinated patients (154 controls and 51 cases) had received Fluad (MF59-adjuvanted trivalent vaccine administered to people ≥65 years). Fluarix Tetra and Vaxigrip Tetra (quadrivalent vaccines administered to people of all ages) were received by 29.3% (83 controls and 18 cases) and 9.9% (20 controls and 14 cases), respectively. Information on vaccine brand was missing for five patients. All patients who received two doses of a vaccine (i.e., were <9 years old) received the same brand for both doses.

### 3.4. Vaccine Effectiveness

When all seasonal vaccines were considered, the estimated VE against any type A influenza was 40.5% (95% CI = 18.7–56.4%), after adjusting for age and presence of chronic conditions (Table 3).

Adjusted VE against subtype A(H1N1)pdm09 influenza was 55.0% (95% CI = 34.5–69.1%) after adjusting for sex, lag time, and month of SARI onset. Adjusted VE against A(H3N2) influenza was 2.5% (95% CI = −50.0–36.7%) after adjusting for age, sex, presence of chronic conditions, month of SARI onset, and number of GP visits in the last 12 months. However, the 95% CI around this point estimate was wide and included zero, suggesting that seasonal vaccination was not effective in preventing SARI caused by subtype A(H3N2) influenza.

**Figure 2:**
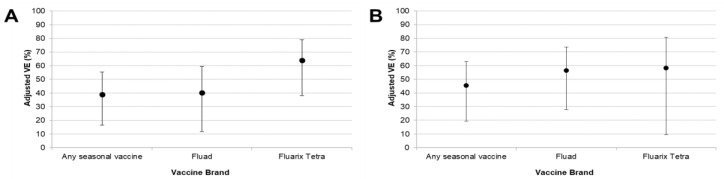
Adjusted estimates of VE (95% CI) against all type A influenza (**A**) and subtype A(H1N1)pdm09 (**B**) stratified by vaccine brand. Abbreviations: CI, confidence interval; VE, vaccine effectiveness; N.B. Vaxigrip Tetra is not included because of low patient numbers receiving this vaccine brand.

When stratified by vaccine brand, both Fluad and Fluarix Tetra vaccines showed effectiveness against type A influenza overall and subtype A(H1N1)pdm09 (Figure 2). In line with results for any seasonal vaccine, neither brand demonstrated VE against subtype A(H3N2) influenza. Estimates of VE for Vaxigrip Tetra were negative, with very wide 95% CI. This was likely due to the low number of cases and controls receiving this vaccine brand.

**Figure 3:**
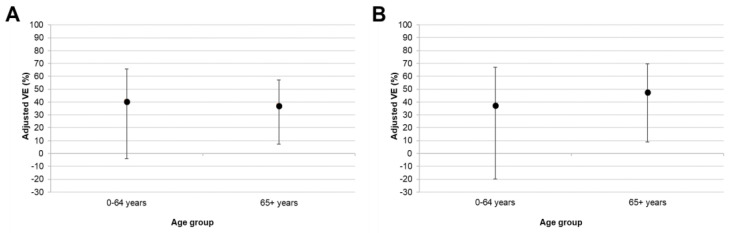
Adjusted estimates of VE (95% CI) against all type A influenza (**A**) and subtype A(H1N1)pdm09 (**B**) stratified by age group.

Stratification by age group (0–64 years, ≥65 years) gave similar point estimates of VE against all type A influenza in both groups (Table 4 and Figure 3).

When only A(H1N1)pdm09 was considered, the point estimate of VE was higher in the ≥65 age group. However, 95% CI around these estimates were extremely wide and, in the 0–64 years group, included zero. This was likely due to the low vaccine coverage in this age group (6.8%). The point estimate of VE against A(H3N2) influenza suggested a low effectiveness in the 0–64 years group, in slight contrast to the adjusted results across all age groups (Table 3). However, we cannot draw any meaningful conclusions from this result, given that the 95% CI included zero and vaccine coverage was low in this group.

## 4. Discussion

A(H1N1)pdm09 and A(H3N2) influenza viruses were isolated among patients with SARI in the five hospitals participating in this study during the 2018/19 influenza season. Subtype A(H1N1)pdm09 viruses were more common than A(H3N2). Type B influenza was uncommon, with only one recorded case in the study sample. This is in line with previously reported findings for the 2018/19 season in people with laboratory-confirmed influenza in Italy [10,11], Europe [12,13], and North America [14,15]. Vaccine coverage in our sample was comparable to the coverage reported in the general Italian population for the 2018/19 season, both overall and across age groups [7].

Our results indicate that seasonal influenza vaccination was effective in preventing SARI caused by type A influenza. However, stratification by virus subtype suggested that seasonal vaccination was not effective against A(H3N2) influenza, while the effectiveness was moderate (~40%) against A(H1N1)pdm09. These results are comparable to findings from wider samples of patients with laboratory-confirmed influenza in primary care, which showed moderate VE against A(H1N1)pdm09 and low/no VE against A(H3N2) in the 2018/19 season in Italy [10] and across Europe [13]. However, these studies also demonstrated moderate effectiveness against A(H3N2) influenza in people ≥65 years, which was not seen in our analysis. Interim results from a study related to the 2018/19 influenza season in primary care in Southern Italy (Sicily) estimated an adjusted VE against any type A influenza (44.0%; 95% CI = 11.2–64.7%), which is similar to our findings [11]. On the other hand, in the same study, a moderate VE against A(H3N2) influenza was also reported across all age groups (40.7%; 95% CI = −1.0–65.3%), in contrast to our findings. Interim results from Canada showed high VE against A(H1N1)pmd09 influenza in primary care (with low circulation of the A(H3N2) strain) [14]. The adjusted VE of 91% (95% CI = 67–98%) reported in this study was much higher than the percentage seen in our analysis.

The differences between our results and those of other studies in the 2018/19 season in the Northern hemisphere could be explained by differing study settings (primary vs. secondary care). Overall, it appears that there was a degree of mismatch between seasonal vaccines and circulating A(H3N2) influenza viruses. Analysis using hemagglutinin assays has demonstrated that A(H3N2) viruses belonging to clades 3C.2a and 3C.3a were poorly recognised by antisera in the 2018/19 vaccines used in the Northern hemisphere. In contrast, the majority of A(H1N1)pdm09 viruses characterised in Europe were antigenically similar to the virus used to produce 2018/19 vaccines [16]. VE against A(H3N2) influenza was also low in the 2016/17 and 2017/18 seasons across Europe, which was attributed to vaccine mismatch [8].

In our study, cases and controls were similar, except for a significant difference in terms of smoking status (*p* = 0.000) and presence of a chronic condition (*p* = 0.015). This is likely because people who smoke daily or have an underlying chronic condition are likely to be more susceptible to the infection and, therefore, are more likely to test positive for influenza.

Our results suggest that Fluad (MF59-adjuvanted trivalent vaccine) and Fluarix Tetra (quadrivalent vaccine) brands were effective against A(H1N1)pdm09 influenza. Estimates of VE for Vaxigrip Tetra were negative, with very wide 95% CI, likely due to the fact that the number of cases and controls receiving this vaccine brand was too low to give an accurate VE estimate. The point estimates of adjusted VE against type A influenza overall suggested that Fluarix Tetra vaccines could potentially be more effective than Fluad vaccines. However, the 95% CI around these estimates had a substantial overlap, and the effect became less pronounced when only A(H1N1)pmd09 influenza was considered. Furthermore, as Fluad is only administered to people ≥65 years, results across all age groups are not directly comparable. Therefore, it is not possible to draw a conclusion as to whether one vaccine brand or type was more effective than the other.

Point estimates of adjusted VE in the 0–64 and ≥65 age groups suggested that VE against type A influenza overall was similar for patients of different ages. When only A(H1N1)pmd09 influenza was considered, results suggested that VE could be higher in the ≥65 age group. However, wide overlapping 95% CI means that this result should be interpreted with caution. In the ≥65 age group, adjusted VE against A(H1N1)pdm09 was around 48%. This is comparable to previously reported estimates of VE against severe influenza requiring hospitalisation in this age group during the 2017/18 season (data on hospitalised patients are not yet available for the 2018/19 season) [8,9].

Our study has some limitations. First, the study may have been underpowered for comparisons between vaccine brands. In particular, the number of cases receiving Fluarix Tetra and Vaxigrip Tetra vaccines were low (18 and 14, respectively). Therefore, these results should be interpreted with caution. Additionally, for VE estimation, we had to stratify patients into two age groups (0–64 and ≥65 years) due to low vaccine coverage in the younger age group of patients. This means that our results could not be compared as readily with previously reported studies, which took into account more age groups, and the VE among children in our study remains unclear.

## 5. Conclusions

Seasonal influenza vaccines received by patients admitted in the study centres in the 2018/19 season were moderately effective in preventing laboratory-confirmed SARI caused by A(H1N1)pdm09 influenza viruses. Vaccines were not effective in preventing SARI caused by A(H3N2) influenza. This is in line with findings in previous seasons and suggests a mismatch between the vaccine and circulating A(H3N2) influenza.

## Figures and Tables

**Table 1:** Comparison of patient characteristics for cases and controls.

Characteristic	Controls (*n* = 1167)	Cases (*n* = 500)	*p*-Value
Sex = male	664 (56.9%)	284 (56.8%)	0.970
Mean age (years) ± SD	35.5 ± 34.8	35.3 ± 32.9	0.920
Age group			0.124
0–17	588 (50.4%)	244 (45.8%)	
18–64 years	182 (15.6%)	108 (21.6%)	
65+ years	397 (34.0%)	148 (29.6%)	
Mean number of GP visits in past year ± SD	2.26 ± 2.12	1.96 ± 2.12	0.015 *
Mean number of hospitalisations in past year ±SD	0.98 ± 1.33	0.92 ± 1.50	0.266
Smoking status			0.000 ***
Never smoked	781 (66.9%)	327 (65.4%)	
Ex-smoker	140 (12.0%)	36 (7.2%)	
Occasional smoker	69 (5.9%)	18 (3.6%)	
Daily smoker	177 (15.2%)	119 (23.8%)	
Presence of chronic condition	522 (44.7%)	256 (51.2%)	0.015 *
Lung disease	249 (21.3%)	109 (21.8%)	
Cardiovascular disease	296 (25.4%)	141 (28.2%)	
Diabetes	144 (12.3%)	42 (8.4%)	
Renal disease	77 (6.6%)	24 (4.8%)	
Cancer	65 (5.6%)	19 (3.8%)	
Liver disease	22 (1.9%)	9 (1.8%)	
Immunodeficiency	15 (1.3%)	6 (1.2%)	
Obesity	35 (3.0%)	19 (3.8%)	
Anaemia	22 (1.9%)	10 (2.0%)	
Dementia	35 (3.0%)	11 (2.2%)	
Rheumatic disease	25 (2.1%)	8 (1.6%)	

Abbreviations: GP, general practitioner; SD, standard deviation; * *p* ≤ 0.05, *** *p* ≤ 0.001.

**Table 2:** Vaccine coverage overall and by age group.

Age Group	No. People Vaccinated (%)		
	Controls	Cases	Total
All ages	260 (22.3%)	85 (17.0%)	345 (20.7%)
0–17 years	17 (2.9%)	10 (4.1%)	27 (3.2%)
18–64 years	36 (19.8%)	13 (12.0%)	49 (16.9%)
65+ years	207 (52.1%)	62 (41.9%)	269 (49.4%)

**Table 3:** Crude and adjusted estimates of vaccine effectiveness (VE), overall and stratified by vaccine brand.

Influenza Subtype	VE (95% CI)							
	Any Seasonal Vaccine (*n* = 260 Controls, 85 Cases)		Fluad (*n* = 154 Controls, 51 Cases)		Fluarix Tetra (*n* = 83 Controls, 18 Cases)		Vaxigrip Tetra (*n* = 20 Controls, 14 Cases)	
	Crude	Adjusted	Crude	Adjusted	Crude	Adjusted	Crude	Adjusted
All type A	28.5 (6.3 to 45.5)	40.5 (18.7 to 56.4)	27.6 (−14.1 to 48.3)	40.2 (11.9 to 59.4)	52.6 (20.1 to 71.9)	63.2 (36.8 to 78.5)	−52.99 (−205.9 to 23.5)	−28.1 (−159.4 to 36.7)
A(H1N1)pdm09	54.4 (34.0 to 68.5)	55.0 (34.5 to 69.1)	60.0 (34.7 to 75.5)	60.9 (35.7 to 76.2)	66.3 (29.8 to 83.8)	66.3 (29.4 to 83.9)	−20.45 (−169.0 to 46.1)	−18.7 (−169.3 to 47.6)
A(H3N2)	−54.5 (−122.8 to −7.1)	2.5 (−50.0 to 36.7)	−69.7 (−162.5 to −9.7)	−17.0 (−98.2 to 30.9)	−0.8 (−105.0 to 50.5)	42.9 (−20.8 to 73.0)	−120.7 (−443.9 to 10.4)	−49.8 (−294.9 to 43.2)

Abbreviations: CI, confidence interval; VE, vaccine effectiveness.

**Table 4:** Adjusted estimates of VE, overall and stratified by age group.

Influenza Subtype	Adjusted VE (95% CI)	
	0–64 Years (*n* = 770 Controls, 352 Cases)	65+ Years (*n* = 397 Controls, 148 Cases)
All type A	40.4 (−3.8 to 65.8)	37.1 (7.3 to 57.3)
A(H1N1)pdm09	37.2 (−19.7 to 67.1)	47.5 (8.9 to 69.7)
A(H3N2)	23.4 (−7.2 to 66.0)	−2.7 (−0.74 to 39.4)

Abbreviations: CI, confidence interval; VE, vaccine effectiveness.
